# Machine learning model demonstrates stunting at birth and systemic inflammatory biomarkers as predictors of subsequent infant growth – a four-year prospective study

**DOI:** 10.1186/s12887-020-02392-3

**Published:** 2020-10-30

**Authors:** Elizabeth Harrison, Sana Syed, Lubaina Ehsan, Najeeha T. Iqbal, Kamran Sadiq, Fayyaz Umrani, Sheraz Ahmed, Najeeb Rahman, Sadaf Jakhro, Jennie Z. Ma, Molly Hughes, S. Asad Ali

**Affiliations:** 1 School of Medicine, University of Virginia, Charlottesville, VA USA; 2 Children’s Hospital of Pittsburgh, University of Pittsburgh Medical Center, Pittsburgh, PA USA; 3 Department of Paediatrics and Child Health, Aga Khan University, Stadium Road, P. O. Box 3500, Karachi, 74800 Pakistan; 4 Department of Public Health Sciences, University of Virginia, Charlottesville, VA USA; 5 Department of Medicine, University of Virginia, Charlottesville, VA USA

**Keywords:** Infant growth, Systemic inflammatory biomarkers, Growth predictors

## Abstract

**Background:**

Stunting affects up to one-third of the children in low-to-middle income countries (LMICs) and has been correlated with decline in cognitive capacity and vaccine immunogenicity. Early identification of infants at risk is critical for early intervention and prevention of morbidity. The aim of this study was to investigate patterns of growth in infants up through 48 months of age to assess whether the growth of infants with stunting eventually improved as well as the potential predictors of growth.

**Methods:**

Height-for-age z-scores (HAZ) of children from Matiari (rural site, Pakistan) at birth, 18 months, and 48 months were obtained. Results of serum-based biomarkers collected at 6 and 9 months were recorded. A descriptive analysis of the population was followed by assessment of growth predictors via traditional machine learning random forest models.

**Results:**

Of the 107 children who were followed up till 48 months of age, 51% were stunted (HAZ < − 2) at birth which increased to 54% by 48 months of age. Stunting status for the majority of children at 48 months was found to be the same as at 18 months. Most children with large gains started off stunted or severely stunted, while all of those with notably large losses were not stunted at birth. Random forest models identified HAZ at birth as the most important feature in predicting HAZ at 18 months. Of the biomarkers, AGP (Alpha- 1-acid Glycoprotein), CRP (C-Reactive Protein), and IL1 (interleukin-1) were identified as strong subsequent growth predictors across both the classification and regressor models.

**Conclusion:**

We demonstrated that children most children with stunting at birth remained stunted at 48 months of age. Value was added for predicting growth outcomes with the use of traditional machine learning random forest models. HAZ at birth was found to be a strong predictor of subsequent growth in infants up through 48 months of age. Biomarkers of systemic inflammation, AGP, CRP, IL1, were also strong predictors of growth outcomes. These findings provide support for continued focus on interventions prenatally, at birth, and early infancy in children at risk for stunting who live in resource-constrained regions of the world.

**Supplementary information:**

The online version contains supplementary material available at 10.1186/s12887-020-02392-3.

## Background

Stunting affects up to one-third of the children in low-to-middle income countries (LMICs) [1]. It is indicative of a failure to achieve genetic potential for height (more than two standard deviations [SD] below the World Health Organization international standards for growth) [2, 3]. Long-term devastating consequences of stunting have been reported which include permanent cognitive impairments, oral vaccine response failure, and diminished immunocompetence [1, 4]. It further accounts for 1.2 million deaths per year among children under 5 years of age [1]. Global income has been estimated to increase by $176.8 billion per year if linear growth failure is eliminated [5]. Linear growth improvement has been reported by previous studies to be refractory to nutritional interventions [6, 7]. This prompts the need to explore whether growth eventually improves (e.g. at 48 months of age) and individuals who are at risk. This will enable eradication of the factors leading to stunting and also warrants well-designed trials to elucidate any and all food-based interventions that might have growth-promoting potential [6].

The age of stunting has direct implications for the progression of growth, as well as the timing and nature of appropriate interventions. Intrauterine growth restriction and small size at birth are strongly associated with risk of stunting at 24 months of age [8]. Most relevant studies have shown that major linear growth failure occurs in the first 48 months of life and beyond this age catch-up growth is rare due to a lack of change in nutrition or environment for older children [9, 10]. A large body of evidence suggests that the first 1000 days from conception is a critical window in which interventions to address malnutrition will be most effective; however, little is known about the impact on linear growth of nutritional interventions in children greater than 2 years of age [6].

The early identification of at-risk infants is critical for early intervention and prevention of subsequent morbidity. Previous studies have shown increased concentrations of inflammatory biomarkers and decreased concentrations of anabolic growth factors such as insulin-like growth factor-1 (IGF-1) to be associated with stunting [4]. Such studies often utilize parametric methods in their data analyses even though nonparametric machine learning-based approaches, such as random forests, frequently outperform parametric approaches in studies with a larger number of variables than observations [11]. Further, with the need for reliable methods to characterize different growth patterns [12], random forest models are robust to overfitting specially if hyperparameters are tuned which increases their applicability in terms of being able to fit more than just a particular set of data [13]. These random forest models are often using for biomedical research which involves multiple variables such as biomarkers that can predict ovarian cancers [14], neuroimaging and biological data for patients with Alzheimer’s disease [15], genes identified via microarray for various diseases [16], among others. This led to the use of random forest model-based approach for predicting subsequent infant growth in our study. It has also been reported that growth characterization models using z-scores were superior in terms of accurate fits compared to fitting model to the original scale for length or height measurement [12] due to which we utilized Height for Age z-scores (HAZ) for our model.

The aim of our study was to investigate patterns of growth in infants up through 48 months of age to assess whether there were improvements, as well as potential predictors of growth such as systemic biomarkers and anthropometric measurements taken at birth.

## Methods

### Data collection

Initially data was collected part of a 4-year prospective parent study for children up till 24 months of age where researchers at Aga Khan University in Pakistan collected data for 380 children from the rural village of Matiari, Sindh, Pakistan [17]. For the purpose of this sub-study, researchers revisited the patients in order to obtain additional consent for anthropometric measurements to be obtained at 48 months of age as the primary end-point. Based on this, the variables utilized for this manuscript were anthropometric measurements collected from birth through the twenty fourth month of life (as part of parent study) and then additionally collected at 48 months of age (based on additional consent acquired for this sub-study) along with the associated demographic and serologic laboratory test data. All anthropometric measurements were converted into z-scores using the World Health Organization child growth standards. Children who did not meet growth and nutritional requirements were subject to additional investigations and interventions as part of the parent study.

### Ethics approval

This study was approved by the Ethical Review Committee of Aga Khan University in Karachi, Pakistan; written informed consent was obtained from parents and/ or guardians.

### Analysis overview

Our analysis was focused on: (1) descriptive study population characterization; and, (2) identification of subsequent growth predictors via random forest analysis using the anthropometric measurements and biomarker levels collected in the first year of life. Visualizations used to aid descriptive analysis included scatter plots and spaghetti plots. Random forests analysis identified predictors of growth via classification and regression. With the aid of additional visualizations, these results were also used to rank the predictors of linear growth at 20 months of life. The predictability of the top 35 variables was then estimated using a linear model. Data preparation, modeling, and analysis were all completed using the Python coding language in Jupyter Notebook, an open-source development environment. The detailed methods for descriptive and random forest analysis are provided below.

### Descriptive analysis

Data exploration initially focused on 48 month outcomes. Of the original cohort (*n* = 380), which was followed for 24 months, 112 infants from the same cohort participated in the follow-up study up till 48 months of age. Out of the 112, 107 infants had sufficient anthropometric data to be included in analysis. Stunting and growth failure in this study were evaluated using HAZ. Stunting was defined as HAZ two standard deviations (SD) below the mean (HAZ < − 2).

Mean HAZ was calculated across the follow-up population at three time points: (1) at enrollment (< 1 month of age); (2) at 18 months of age; and, (3) at 48 months of age. Patterns in the distribution of stunting across both sexes were examined at each point. Then the influence of location was examined including any patterns associated within the village or Union Council. Subsequently, based on the same three time points for mean HAZ calculation, the population was divided into subgroups based on stunting (HAZ < − 2) status at each of the study visits. This allowed for the examination of general growth trends using categorical variables of stunted versus not-stunted. Growth trends were also evaluated using linear regression plots and correlation coefficients (r; using Pearson correlation), with x and y values based on the raw HAZ of the individual children at each of the three time points.

In order to assess the change in growth, this study further examined growth trends based on the changes in HAZ over time (delta HAZ). Delta HAZ were calculated by subtracting the z-scores at 18 and 48 months from those given at the first clinic visit. With a slightly smaller subset of 101 children, a spaghetti plot was used to identify growth patterns in the follow-up cohort using monthly HAZ measurements from the first 18 months of life, as well as from the 48-month follow-up visit. Relevant delta HAZ outliers were then highlighted using different colors based on whether their delta HAZ was notably positive or negative which also led to spaghetti plot-based visualization of the growth trends of the children over time.

### Random Forest models

This study’s final models were designed to be interpretable, with a significantly reduced set of predictors (details in Supplementary Methods as part of Additional File 1). All models were created using sklearn’s Random Forest Classification and Regressor packages. These python packages utilized for random forest analyses have been developed by Scikit-learn and are state-of-the-art implementation packages created to maintain an easy-to-use interface tightly integrated with the Python language [18]. List of biomarkers and cytokines included for the random forest analyses is provided in Additional File 2.

Identification of variables that were highly predictive of stunting was approached in two ways: (1) with a random forest classification model using stunted versus not-stunted as outcomes; and, (2) random forest regression using HAZ at 18 months as the outcome variable. For both approaches, an 80–20 test-train split was used. To minimize bias, children who participated in the follow-up were divided randomly across training and testing groups. All numeric variables were scaled using sklearn’s min_max_scaler.fit_transform() function.

In classification, random forest hyperparameters were optimized using a grid search. This grid search included “n” estimators ranging from 5 to 300, max features ranging from 25 to 106 (all features), max depths ranging from 5 to “None,” minimum sample splits of 2 and 4, and minimum sample leaves of 1 and 2. The grid search comprised 300 iterations with 5-fold cross validation. Optimized parameters included max depth at 100, max features at 106 (all features), “n” estimators at 80, minimum sample leaf number at 2, and minimum sample split at 4. All other hyperparameters were set to the function’s default. Feature importance results were then extracted, and the top 35 features of the forest were plotted using a labeled bar chart with lines over each bar representing the inter-tree variability of each feature (explained in non-technical terms as part of Supplementary Methods; Additional File 1).

In regression, performance of a baseline model using all default hyperparameters was compared to that of a model using hyperparameters optimized with a similar grid search to that noted above. Performance was relatively comparable and highly dependent on random state, so the baseline random forest regressor model was used for feature analysis. Again, the top 35 features of importance were extracted and plotted. These were then compared to the outputs of the classification model.

## Results

### Descriptive analysis

**Fig. 1:**
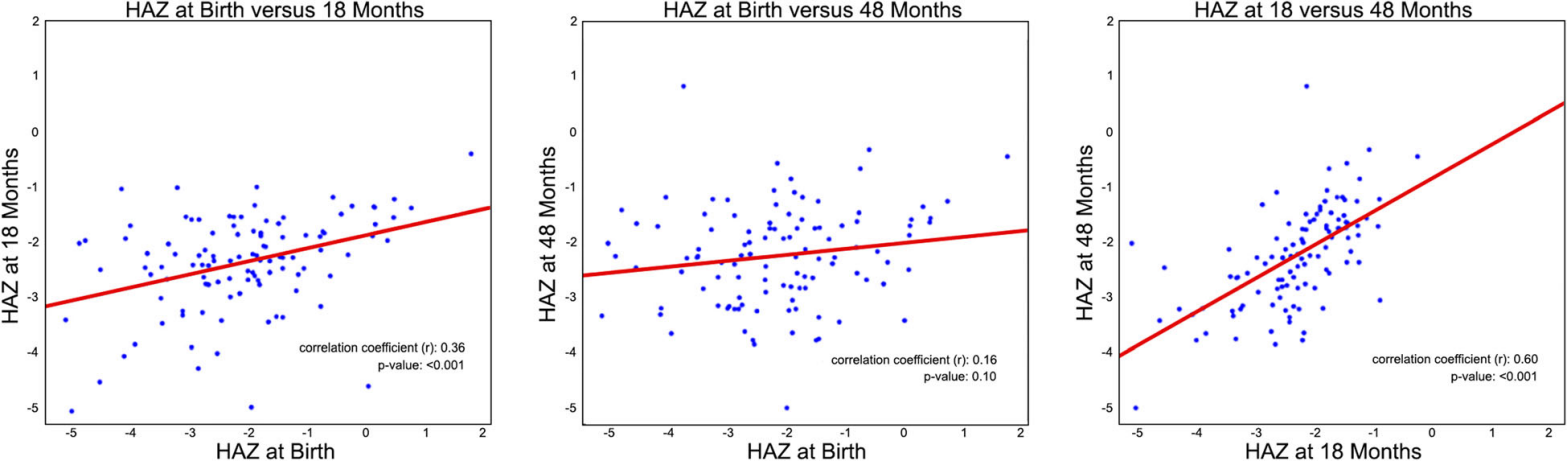
Comparison of Height for Age z-scores at birth, 18 months, and 48 months. Key: HAZ - Height for Age z-score

A total of 112 children who were followed up to 48 months and 107 out of those with sufficient anthropometric data were included from the follow-up cohort during initial descriptive analysis (Tables 1 and 2). 46% were males and 51% were stunted at birth (51% male among those stunted at birth). At 18 months of age, the percentage of children with stunting increased to 64% while the male percentage of this subgroup decreased to 46%. By the 48-month visit, the percentage of stunted children had dropped back down to 54% with only 40% of these being male. Using scatter plots and linear fit regression lines, HAZ at birth, 18 months, and 48 months were compared (Fig. 1). While HAZ at birth were weakly linearly correlated with HAZ at either 18 months or 48 months (*r* = 0.376; *p* = 0.0001 and *r* = 0.162; *p* = 0.0954, respectively). HAZ at 18 months showed a stronger positive linear correlation with HAZ at 48 months (*r* = 0.604; *p* < 0.0001).

**Table 1:** Patient and Maternal Characteristics of the 107 infants followed till 48 months of age

Characteristics	Frequency (%)
Gender, Male (%)	46
Preterm Birth (%)	52^a^
Advanced Maternal Age (%)	64^a^
Breastfed soon after birth (%)	98^a^
Literate Mother (%)	13^a^

^a^ – values missing for 6 children

**Table 2:** Anthropometric measurement based WHZ, HAZ and WAZ of the 107 infants followed till 48 months of age

Anthropometric Measurements	Birth (mean, ± SD)	18 months of age (mean, ± SD)	48 months of age (mean, ± SD)
WHZ	−0.25, ± 1.30	−0.81, ± 0.92	− 0.66, ± 0.89
HAZ	−2.09, ± 1.35	−2.37, ± 0.87	− 2.11, ± 0.87
WAZ	−1.68, ± 1.17	−1.73, ± 0.91	− 1.73, ± 0.83

Key: *WHZ* Weight for Height z-score; *HAZ* Height for Age z-score; *WAZ* Weight for Age z-score; *SD* Standard Deviation

The 107 children were examined by dividing them into groups based on their stunting status at the same time points (birth, 18 months, and 48 months). As shown in Table 3, of the 107 children, 20 children consistently had HAZ above − 2 regardless of age while 30 children who began stunted (HAZ < − 2) remained stunted at their 18 and 48 follow ups. Of the 55 children who were stunted at birth, 12 were not stunted by 18 months (and remained in the normal HAZ range) and an additional 11 were not stunted by 48 months of age. Of the 52 children who were not stunted at birth, 21 fell into the stunted range by 18 months and remained stunted at their 48 month follow-up and an additional 5 children fell into the stunted range between their 18-month and 48-month checks.

**Table 3:** Groups based on stunting status and follow up time points of the 107 infants followed till 48 months of age

Birth	18 months of age (mean, ± SD)	48 months of age (mean, ± SD)	Number of Children (total ***n*** = 107)
Not Stunted	Not Stunted	Not Stunted	20
		Stunted	5
	Stunted	Not Stunted	6
		Stunted	21
Stunted	Not Stunted	Not Stunted	12
		Stunted	2
	Stunted	Not Stunted	11
		Stunted	30

Key: *SD* Standard Deviation

**Fig. 2:**
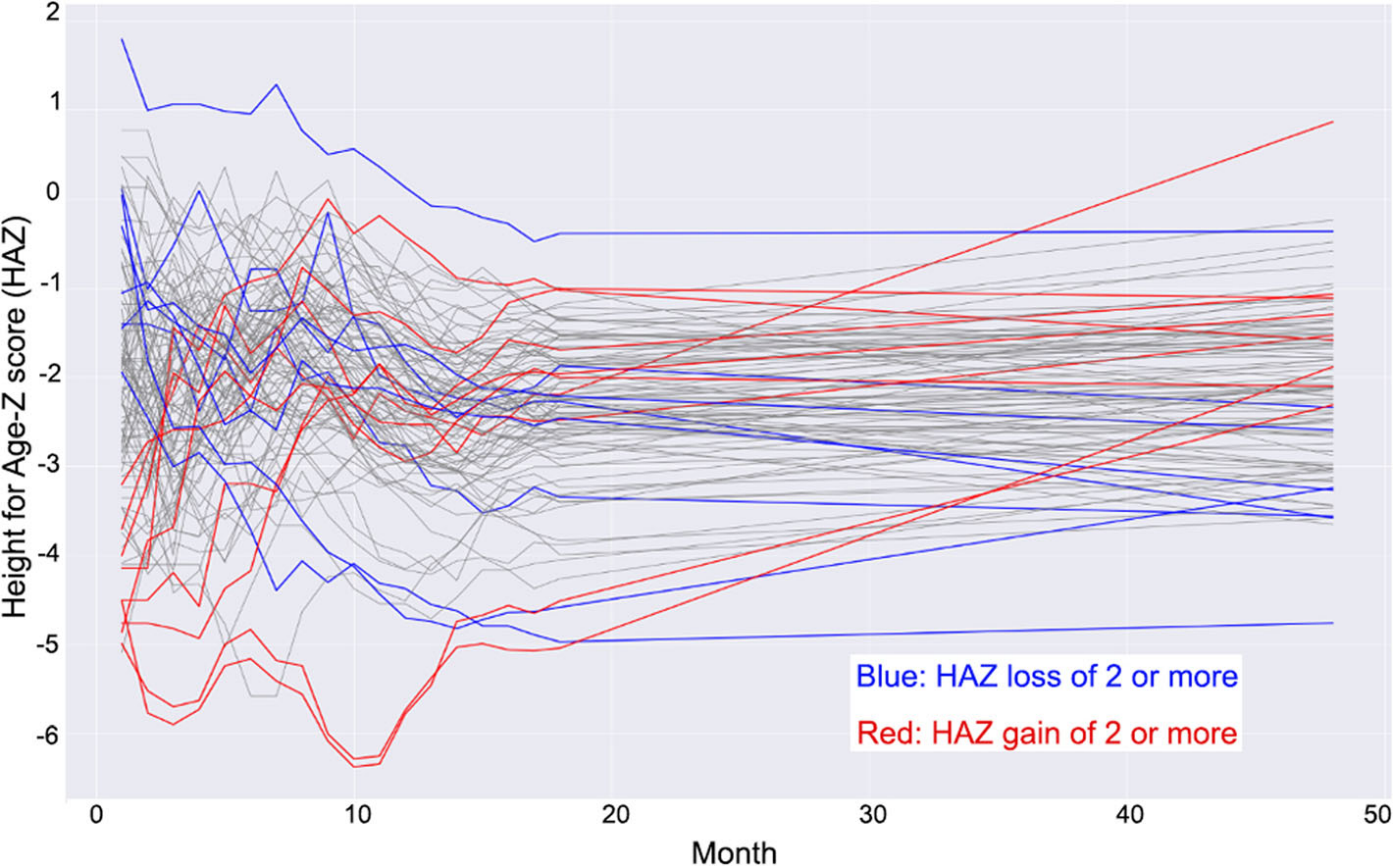
Spaghetti plot for growth trends of infants with follow up 48 months of age (6 patients excluded due to insufficient anthropometric data). Key: HAZ - Height for Age z-score

Using a more granular approach in order to better visualize growth trends spaghetti plots were generated (Fig. 2). Spaghetti plots included 101 out of the 107 children as the excluded six children had less frequent visits in the first 2 years of the study. As shown in the plot, there were 9 children with delta HAZ above 2 (z-scores increased by at least 2 points) and 8 children with delta HAZ under − 2 (z-scores decreased by at least 2 points). All those with a positive change in z-score of 2 or more were noted to be stunted at their first study visit. Conversely, all those with a loss in their HAZ of 2 or more were notably not stunted at their first study visit. Notably, most of the children who dropped in their HAZ experienced most losses in the first 2 years while children who grew well (HAZ normal range) experienced highest gains between the 18 and 48 month visit. Across the entire follow-up cohort, most children appear to gain little between the 18-month and 48-month visits, though generally remaining around the same z-score.

### Random Forest models

**Fig. 3:**
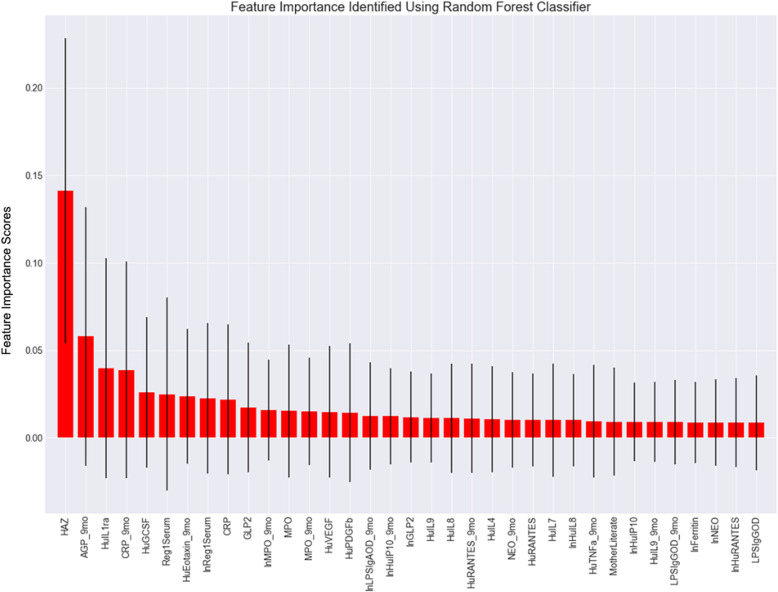
Random Forest Classifier based relative feature importance for the top 35 features predicting subsequent infant growth (hyperparameter optimization done via a grid search with cross-validation – y-axis shows feature importance scores which do not have a specific unit). Key (alphabetical): AGP – Alpha- 1-acid Glycoprotein; CRP – C-reactive Protein; GLP2 – Glucagon-like peptide 2; HAZ - Height for Age z-score; HuEotaxin – Human Eotaxin; HuGCSF – Human Granulocyte-colony stimulating factor; HuIL4 – Human Interleukin-8; HuIL7 – Human Interleukin-7; HuIL8 – Human Interleukin-8; HuIL9 – Human Interleukin-9; HuILra – Human Interleukin-1 Receptor Antagonist; HuIP10 – Human Interferon gamma-induced protein 10;HuPDGFb – Human Platelet Derived Growth Factor Subunit B; HuRANTES – Human RANTES (CCL5; C-C Motif Chemokine Ligand 5); HuTNFa – Human Tumor necrosis factor-α; HuVEGF – Human Vascular Endothelial Growth Factor; LPSIgAOD – Lipopolysaccharide IgA Optical Density; LPSIgGOD – Lipopolysaccharide IgG Optical Density; MotherLiterate – Literacy Status of the Mother; MPO – Myeloperoxidase; NEO – Neopterin; Reg1Serum – Serum Regenerating Gene 1β. Note: ‘ln’ before a variable refers to natural logarithm, ‘_9mo’ after a variable refers to the biomarker being collected at 9 months of follow-up, variables without mention of a time frame were collected at birth

After random forest model implementation and hyperparameter optimization, relative feature importance was extracted and plotted for the top 35 features (Fig. 3). As shown, the only feature identified with high importance and an inter-tree variability line which did not cross zero was the raw HAZ calculated using anthropometry done at birth. Other relatively important features in this forest included Alpha- 1-acid Glycoprotein (AGP) and C-Reactive Protein (CRP) biomarker levels at 9 months, as well as interleukin-1 (IL1); however, all of these demonstrated significant variability with some trees assigning these features low to zero importance. With optimization, this random forest model predicted stunting at 18 months of age in the set-aside testing set with 78% accuracy.

**Fig. 4:**
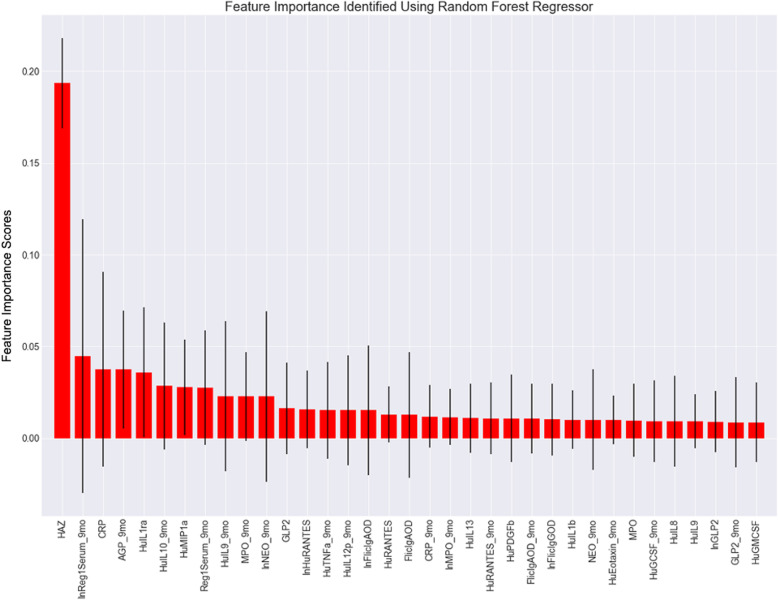
Random Forest Regressor based relative feature importance for the top 35 features predicting subsequent infant growth (y-axis shows feature importance scores which do not have a specific unit). Key (alphabetical): AGP – Alpha- 1-acid Glycoprotein; CRP – C-reactive Protein; GLP2 – Glucagon-like peptide 2; HAZ - Height for Age z-score; HuEotaxin – Human Eotaxin; HuGCSF – Human Granulocyte-colony stimulating factor; HuIL4 – Human Interleukin-8; HuIL7 – Human Interleukin-7; HuIL8 – Human Interleukin-8; HuIL9 – Human Interleukin-9; HuILra – Human Interleukin-1 Receptor Antagonist; HuIP10 – Human Interferon gamma-induced protein 10;HuPDGFb – Human Platelet Derived Growth Factor Subunit B; HuRANTES – Human RANTES (CCL5; C-C Motif Chemokine Ligand 5); HuTNFa – Human Tumor necrosis factor-α; HuVEGF – Human Vascular Endothelial Growth Factor; LPSIgAOD – Lipopolysaccharide IgA Optical Density; LPSIgGOD – Lipopolysaccharide IgG Optical Density; MotherLiterate – Literacy Status of the Mother; MPO – Myeloperoxidase; NEO – Neopterin; Reg1Serum – Serum Regenerating Gene 1β. Note: ‘ln’ before a variable refers to natural logarithm, ‘_9mo’ after a variable refers to the biomarker being collected at 9 months of follow-up, variables without mention of a time frame were collected at birth

Using a similar approach using the random forest regressor package, 35 features were once again identified and plotted (Fig. 4). Although there were a few features other than HAZ in this model with variability lines that did not cross zero, these results were inconsistent across different runs during model development which were due to changes in the random state assigned to each regressor. Although it is notable that the levels of AGP at 9 months of age presented as a strong feature in the results of the classification and regression model along with having reduced variability between the trees of the regressor random forest model. IL1 and CRP at 6 months was also identified as an important feature (compared to IL1 and CRP at 9 months with random forest classification only model).

A baseline random forest regression model using all default hyperparameters and a model using hyperparameters identified with a grid search performed comparably on an unseen testing set. Both models predicted HAZ at 18 months with a mean squared error between 0.7 and 0.8, depending on random state assignments.

Conspicuously, none of the models identified sex, gestational age, or any of the maternal factors as highly important features. The only one of these features to be included in the top 35 of either model was maternal literacy (in the random forest classifier model) but it was of minimal importance across all trees in the forest.

## Discussion

We investigated the serum biomarker and anthropometric predictors of the growth of infants up till 48 months of age among a population from a rural village at an LMIC. A descriptive analysis was followed by the utilization of machine learning-based nonparametric traditional machine learning random forest models which added value to use of such models for answering clinical questions such as predictors of growth. The major results of this work include the following: (1) 51% of the infants were found to be stunted at birth with most of them staying stunted at 48 months of age; (2) a stronger correlation exists between HAZ at 18 and 48 months when compared to the correlation between HAZ at birth and either 18 or 48 months; and, (3) of all the systemic biomarkers and anthropometric measurements, HAZ at birth, AGP, CRP, and IL1 were found to be the strongest predictors of stunting.

Most studies have shown that major linear growth failure occurs in the first 48 months of life; beyond this age, catch-up growth is rare [9, 10]. A large body of evidence suggests that the first 1000 days from conception is a critical window in which interventions to address malnutrition will be most effective but little is known about the impact on linear growth of nutritional interventions in toddlers over the age of 2 years [6]. In our study, HAZ at 18 months had a positive linear correlation with HAZ at 48 months, while HAZ at birth was less strongly correlated with HAZ at either 18 or 48 month of age. The positive correlation of HAZ at 18 and 48 months of age is supported by earlier reporting of minimal levels of catch-up growth from age 2 to 5 years [9]. An increase in HAZ of children with stunting may also be a result of regression to the mean as shown in previous studies [10].

HAZ at birth was shown to be a significantly strong predictor of growth followed by CRP, AGP, and IL1. Much emphasis has been placed on stunting status at birth and overall prenatal helth affecting clinical outcomes in previously published studies including stunting later on in life and poor neurodevelopmental outcomes [19–21]. Such findings highlight the importance of interventions in the early months of life to prevent subsequent stunting and its consequences [20]. It is important to note that nutritional interventions have shown little to no effect with regard to addressing neurodevelopmental outcomes and other long-term consequences if the child’s stunting status is not also addressed [6]. In order to design and construct trials that truly alleviate or reduce the consequences of stunting, the underlying factors that contribute to stunting itself need to be understood.

The serological biomarkers CRP, AGP, and IL1 succeeded stunting at birth in predicting growth among children. CRP and AGP are acute phase proteins stimulated by the release of cytokines such as IL1, IL6, and TNF-α (Tumor necrosis factor-α) [15]. CRP rises and declines rapidly during an acute phase response, whereas AGP rises more slowly (more than 24 h after onset of inflammation) and remains elevated for longer [22–24]. These findings are similar to a previous study by our group. In our previous work, we found significant correlations between flagellin- and lipopolysaccharide-specific Immunoglobulin A (LPS-specific IgA), serum CRP, AGP and Regenerating Gene 1β (Reg1) at 6 months, and myeloperoxidase (MPO) at 9 months. In the previous study, we found that higher anti-LPS IgA levels predicted greater declines in HAZ over the subsequent 18 months of follow-up [25]. In contrast to this prior work, the current work utilizes a machine-learning model to investigate biomarkers as predictors of growth among infants.

Mixed association between inflammation and growth outcomes has also been reported previously. A study done among Zimbabwean infants (an LMIC setting) showed that levels of inflammatory biomarkers (CRP and AGP) measured at 6 weeks, 6 months, 12 months, and 18 months were consistently higher in children with stunting (HAZ < − 2) versus healthy controls (defined as HAZ > − 0.5) at 18 months [26]. Further, among apparently healthy Zimbabwean infants with increased inflammatory biomarkers, the levels of anabolic hormone IGF-1 were low. This finding highlighted the significance of even low-grade inflammation with regard to poor growth outcomes [26]. All findings provide support for continued focus on interventions at birth and early infancy in children at risk for stunting who live in resource-constrained regions of the world.

Knowledge about the biomarkers predictive of stunting is not only important from the perspective of constructing effective interventional trials but also paves the way for understanding the underlying pathology of stunting. The health of pregnant mothers has been shown to effect the infants at birth [27]. It has been reported that stunting begins in utero and continues for at least the first 2 years of postnatal life; the period from conception to a child’s second birthday (the first 1000 days) has therefore been identified as the most critical window of opportunity for interventions [19]. Higher levels of inflammatory markers among infants can be due to ongoing inflammation in the pregnant mothers or infections contracted during early life in the setting of poor sanitation and hygiene.

Several strengths of our study merit mention. Since this is a prospective study, all patients underwent biomarker collection and anthropometric measurements within a similar time frame. We followed a cohort of children for not only up through 24 months, but 48 months of age to assess growth outcomes and predictors and to answer the important questions regarding growth patterns between 2 and 5 years of age. The repeated measurements of length and height allowed our analysis evaluate growth status at birth, 18 months, and 48 months of age along with systemic biomarker levels, allowing us to assess the best predictors of growth beyond 2 years of age. Finally, we utilized a robust machine learning model to perform random forest analysis for the investigation of systemic biomarker and anthropometric growth predictors. Due to the prospective nature of this study, limitations included missing data points for Pearson correlations between known characteristics of participants who failed to return for follow-up or were missing biomarker results because of sample limitations. This also prompted the machine learning model to be designed based on sufficient data available at 18 months of age rather than 48 months of age.

An interesting question for future analysis will be the assessment of in-utero growth and inflammation as potential predictors of subsequent infant growth. Further, investigation of maternal factors, including systemic inflammation, might answer the important unanswered question regarding their role in the growth of the child along with stunting and its prevention.

## Conclusion

We described the growth of infants up through 48 months of age and investigated the potential indicators of subsequent growth. While several of our findings such HAZ at birth, AGP, CRP, and IL1 as predictors of subsequent growth)reiterate previous data, our work solidifies previous assessments of growth through 2 years of age and utilizes a robust a machine learning approach to confirm these measures as predictive of early infant growth patterns. This is significant as it stresses the need to investigate maternal factors leading to stunting. It also highlights specific biomarkers that need to be factored in during construction of future trials targeted towards improvement of growth. These findings provide support for continued focus on interventions at birth and early infancy in children at risk for stunting who live in resource-constrained regions of the world.

## Supplementary information

**Additional file 1.** Supplementary Methods.

**Additional file 2.** List of biomarkers and cytokines included in the random forest model.

## Data Availability

Biomarker data used for the analysis of this study is available as part of the supplementary Additional File 2. Additional data used and/or analyzed during the current study is available from the corresponding authors on a reasonable request.

## Abbreviations

LMIC: Low-to-middle income country
HAZ: Height-for-age z-score
AGP: Alpha- 1-acid Glycoprotein
CRP: C-Reactive Protein
IL1: interleukin-1
IGF-1: insulin-like growth factor-1
WHZ: Weight for Height z-score
HAZ: Height for Age z-score
WAZ: Weight for Age z-score
SD: Standard Deviation
GLP2: Glucagon-like peptide 2
HuEotaxin: Human Eotaxin
HuGCSF: Human Granulocyte-colony stimulating factor
HuIL4: Human Interleukin-8
HuIL7: Human Interleukin-7
HuIL8: Human Interleukin-8
HuIL9: Human Interleukin-9
HuILra: Human Interleukin-1 Receptor Antagonist
HuIP10: Human Interferon gamma-induced protein 10
HuPDGFb: Human Platelet Derived Growth Factor Subunit B
HuRANTES: Human RANTES (CCL5; C-C Motif Chemokine Ligand 5)
HuTNFa: Human Tumor necrosis factor-α
HuVEGF: Human Vascular Endothelial Growth Factor
LPSIgAOD: Lipopolysaccharide IgA Optical Density
LPSIgGOD: Lipopolysaccharide IgG Optical Density
MotherLiterate: Literacy Status of the Mother
MPO: Myeloperoxidase
NEO: Neopterin
Reg1Serum: Serum Regenerating Gene 1β
IL6: Interleukin-6
TNF-α: Tumor necrosis factor-α
