# Supplementary material for: Machine learning model demonstrates stunting at birth and systemic inflammatory biomarkers as predictors of subsequent infant growth – a four-year prospective study
Source: BMC Pediatr. 2020 Oct 30;20:498. doi: 10.1186/s12887-020-02392-3 (PMC7597024; doi:10.1186/s12887-020-02392-3)
Supplement: Supplementary file 1 — Additional file 1. Supplementary Methods. [file 12887_2020_2392_MOESM1_ESM.docx]

**Machine Learning Model Demonstrates Stunting at Birth and Systemic Inflammatory Biomarkers as Predictors of Subsequent Infant Growth – A Four-year Prospective Study.**

**Authors:** Elizabeth Harrison^1,2¶^, Sana Syed^1,3¶^*, Lubaina Ehsan^1^, Najeeha T. Iqbal^3^, Kamran Sadiq^3^, Fayyaz Umrani^3^, Sheraz Ahmed^3^, Najeeb Rahman^3^, Sadaf Jakhro^3^, Jennie Z. Ma^4^, Molly Hughes^5^, S. Asad Ali^3^*

**Affiliations:** ^1^School of Medicine, University of Virginia, Charlottesville, VA, ^2^Children’s Hospital of Pittsburgh, University of Pittsburgh Medical Center, PA, ^3^Department of Paediatrics and Child Health, Aga Khan University, Karachi, Pakistan, ^4^Department of Public Health Sciences, University of Virginia, Charlottesville, VA, ^5^Department of Medicine, University of Virginia, Charlottesville, VA

* co-corresponding authors

^¶^ These authors contributed equally to this work.

**Supplemental Methods**

Our dataset had multiple variables for predicting growth outcomes. Datasets with a large amount of data require methods for selection of important features for outcome prediction. Feature selection is the process of reducing the number of input variables when developing a predictive model such as the one in our study (1, 2). These feature importance scores are further shown to be highly important in building predictive models that are efficient along with providing insight into the variables important for predicting outcomes (1), such as growth in our study.

There are various methods for calculating feature importance which include Random forest, XGBoost, and CART. Random forests are traditional machine learning models that are able to correct for overfitting better than XGBoost and CART for less noisy non-image datasets like ours due to which we selected it for the purpose of our study (3), due to which we selected it for the purpose of our study. Random forests are a learning method for classification, regression and other tasks that operate by constructing a multitude of decision trees at training time and outputting the class that is the mode of the classes (classification) or mean prediction (regression) of the individual trees.

Random forest classification, in laymen terms, suppose within the training set the children with stunting at birth (variable) are more often stunted at 20 months of age (outcome), the random forest will learn this. It will learn other similar variables and their relationship with the outcome. It will keep making decision trees based on the variables and their corresponding relation with the outcome, and finally the variable that gets the majority of the votes (mode of the classes) will be classified as the strongest predictor of the outcome. Random forest regression, rather than the mode of the classes (as used in classification), uses the mean prediction of the individual trees to predict the outcome.

For the purpose of the above mentioned Random forests we used sklearn’s Random Forest Classification and Regressor packages. These packages use python programming language that have been developed by Scikit-learn and are a state-of-the-art implementation package created to maintain an easy-to-use interface tightly integrated with the Python language (4). These packages use the same method by creating multiple decision trees although they do not provide visualization of the trees that were created.

**References**

1. Li J, Cheng K, Wang S, Morstatter F, Trevino RP, Tang J, et al. Feature selection: A data perspective. ACM Computing Surveys (CSUR). 2017;50(6):1-45.

2. Cai J, Luo J, Wang S, Yang S. Feature selection in machine learning: A new perspective. Neurocomputing. 2018;300:70-9.

3. Pink CM. Forensic ancestry assessment using cranial nonmetric traits traditionally applied to biological distance studies. Biological Distance Analysis: Elsevier; 2016. p. 213-30.

4. Pedregosa F, Varoquaux G, Gramfort A, Michel V, Thirion B, Grisel O, et al. Scikit-learn: Machine learning in Python. the Journal of machine Learning research. 2011;12:2825-30.
